# Surgical technique for volar plate, collateral ligament reconstruction, and neglected dislocation of the proximal interphalangeal joint in the fifth finger: A case report

**DOI:** 10.1016/j.ijscr.2025.111258

**Published:** 2025-04-04

**Authors:** Heri Suroto, Yohanes Aprianto Senduk Widodo

**Affiliations:** a Department of Orthopedics and Traumatology, Faculty of Medicine, Universitas Airlangga, East Java, Surabaya, Indonesia; b Department Orthopedics and Traumatology, Dr. Soetomo General Academic Hospital, East Java, Surabaya, Indonesia; c Cell & Tissue Bank-Regenerative Medicine Center, Dr. Soetomo General Academic Hospital, East Java, Surabaya, Indonesia

**Keywords:** Chronic dislocation, Finger dislocation, Joint contracture, Open reduction, Proximal interphalangeal joint

## Abstract

**Introduction and importance:**

Chronic unreduced dislocations of the proximal interphalangeal joint are uncommon and management principles for these injuries have not been defined. The dislocation can be either volar or dorsal and closed reduction is rarely successful due to soft tissue contractures.

**Case presentation:**

A 61-year-old woman presented with a 17-year-old deformity of her left fifth finger, sustained from a bathroom slip. Initially, the patient complained of pain and deformity. After failed of conservative treatment, patient was referred for surgical intervention. Physical examination revealed a dorsal (posterior) deformity. X-ray showed displacement and ulnar deviation of the proximal interphalangeal (PIP) joint of the left fifth finger. The patient underwent operative management with open reduction and radial collateral ligament reconstruction using proximal phalanx intraosseous suturing and volar plate reconstruction. One day after surgery, the patient achieved a passive range of motion (PROM) of 0–80° and an active range of motion (AROM) of the proximal interphalangeal joint (PIPJ).

**Clinical discussion:**

Untreated or unreduced PIPJ dislocations are rare. These injuries are usually caused by hyperextension forces on the finger. Chronic dislocation of the proximal phalanx is uncommon. The stability of the PIPJ is maintained by the volar plate, collateral ligaments, and extensor expansion. Open reduction of persistent PIPJ dislocations can successfully produce a functional range of motion with a stable joint.

**Conclusion:**

A functional range of motion with a stable joint can be achieved as long as the articular cartilage is relatively preserved after surgery.

## Highlights

• Chronic unreduced dislocations of the proximal interphalangeal joint are uncommon.
• These injuries are usually caused by hyperextension forces on the finger.
• Surgical procedure was performed with open reduction and radial collateral ligament reconstruction.
• The reduced PIPJ was augmented by repairing the remnant volar plate.
• Delayed treatment of chronic PIP joint dislocation, may increase the complications.

## 1 Introduction and importance

Chronic dislocation of the proximal interphalangeal joint (PIP) is uncommon and is defined as persistent dislocation or subluxation of the joint due to late presentation or missed finger injury for 17 years or more [1]. Limited literature is available on the management of chronic dislocations.

Various injury patterns for PIP joint dislocation have been described from hyperextension to rotational torque injuries. Different combinations of structures are injured, based on mechanism, including the collateral ligaments, volar plate, and the central slip of the extensor mechanism [2]. Unless there is soft tissue interposition, closed reduction is usually accomplished easily [1].

We aim to describe the surgical technique for open reduction and soft tissue repair of chronic PIP joint dislocations via a zig-zag approach.

## 2 Case presentation

61-year-old female patient presented with a chief complaint of deformity in the fifth finger of the left hand. The patient presents with a chief complaint of deformity in the fifth finger of the left hand since 17 years. This condition occurred after a patient slips in the bathroom. Initially, the patient reported pain, deformity, and an inability to flex the finger. The patient is a medical doctor and can still perform daily activities. After failed of conservative treatment, patient was referred for surgical intervention.

**Fig. 1:**
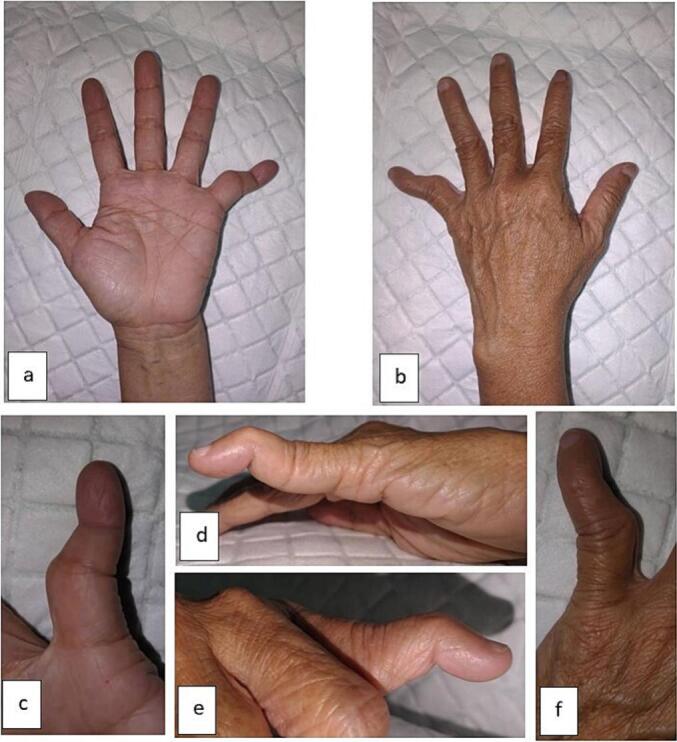
Clinical photograph of the fifth finger of the left hand showing deformity, (a) volar aspect of the left hand, (b) dorsal aspect of the left hand, (c) volar aspect, (d) lateral aspect, (e) medial aspect, (f) dorsal aspect.

**Fig. 2:**
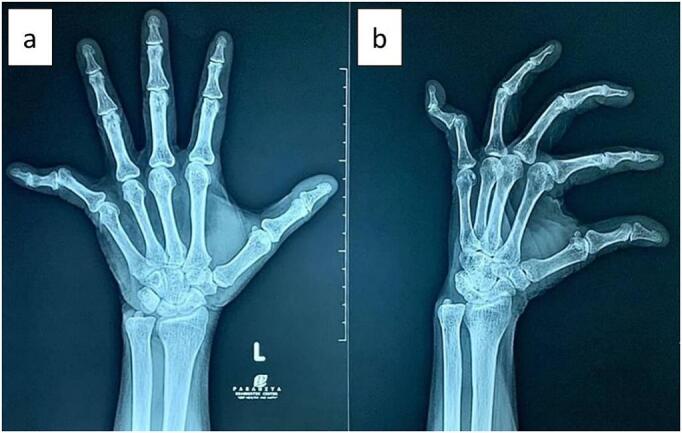
Plain radiograph of the left wrist with (a) posteroanterior and (b) oblique view.

A dorsal (posterior) deformity and ulnar deviation of the PIP joint of the left hand were observed (Fig. 1). The patient was unable to flex her fifth finger. The radiological examination was performed using a plain radiograph initial left wrist with posteroanterior and oblique projections which can be seen in the following Fig. 2.

**Fig. 3:**
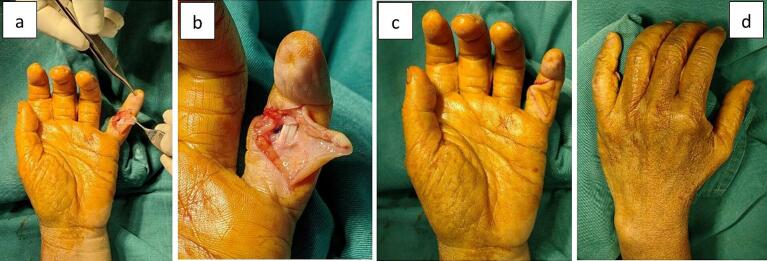
Clinical photograph during operation (a) volar approach, (b) soft tissue exploration, (c) volar aspect after reduced dislocation, (d) dorsal aspect after a reduced dislocation.

After undergoing a series of physical and supportive examinations, the diagnosis of the case was stated as neglected dislocation of the PIP joint in the fifth finger. The patient underwent operative management with open reduction using the volar approach. The incision began in the volar diagonal of the fifth finger. The dislocated PIPJ was reached after identification of the neurovascular bundle and flexor tendon pulley. The volar plate was found to have atrophy and thinning of articular cartilage. The radial collateral ligament was absent. The dislocated PIPJ was reduced but remained unstable. PIPJ reconstruction started with the creation of a bony tunnel at the radio volar proximal of the mid phalanx. Radial collateral reconstruction was performed by suturing nonabsorbable prolene 4.0 through the bony tunnel to the radial side of the proximal phalanx. The reduced PIPJ was augmented by repairing the remnant volar plate to achieve improved stability. Before closing the skin, the stability of the PIPJ was evaluated by passive flexion and extension (Fig. 3).

**Fig. 4:**
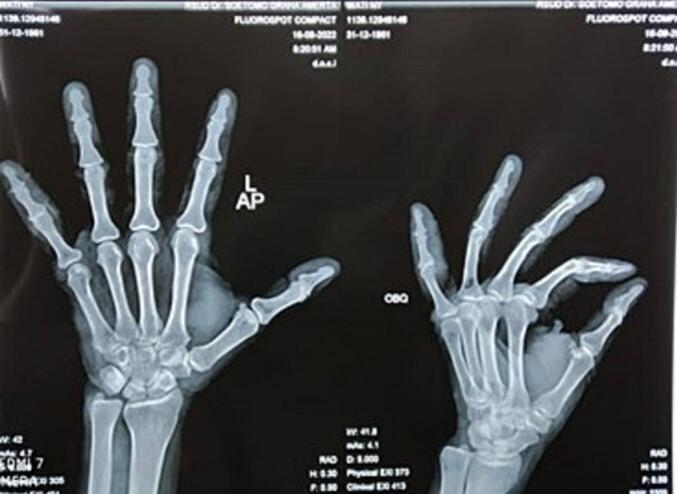
Plain radiograph of the left wrist with (a) posteroanterior and (b) oblique view post operation.

**Fig. 5:**
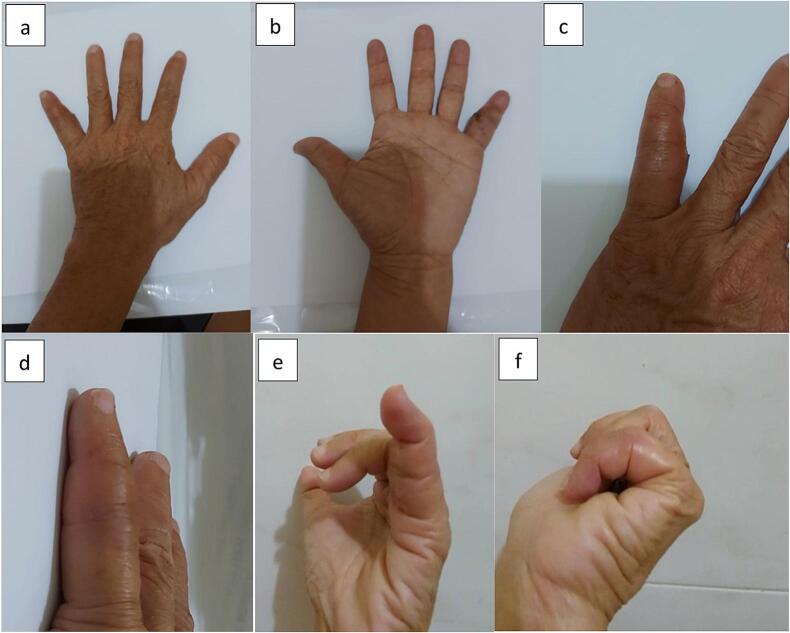
Clinical photograph 1 month post operation. (a) Dorsal aspect of the left hand, (b) volar aspect of the left hand, (c) dorsal aspect, (d) lateral aspect, (e) medial aspect.

After the surgery, the patient did early exercise to prevent stiffness. Post-operative evaluation results showed clinical improvement in ROM (Fig. 4, Fig. 5). One month after surgery the patient could move passive and active PIP joints.

## 3 Discussion

PIP joint dislocations that are not treated or reduced are rare. These injuries are usually caused by hyperextension force on the finger [1]. In normal practice, it is quite rare to develop chronic dislocation of the proximal phalanx, although PIP joint injuries are one of the most common injuries to the hand [3]. Dislocation of the proximal interphalangeal joint usually is usually detected early as it will give a distinct clinical picture. The most common clinical sign is the patient's inability to flex or extend the finger. However, in this case, the dislocation was picked later as the patient presented late for treatment.

The stability of the proximal interphalangeal joint is maintained by volar plate, collateral ligament and extensor expansion. Dorsal dislocation is the most common form. It is associated with volar plate or collateral ligament ruptures [4]. Any laterally deviating force to the tip of the finger will affect the distribution of stress over the collateral ligaments. Significant injury may also disrupt the secondary PIP joint stabilizers [3].

After receiving treatment for a PIP joint injury, several problems may arise. Although joint stiffness and flexion contracture are most common, other complications include re-dislocation, posttraumatic arthritis, chronic swelling, or even permanent functional loss [3]. Given the complex anatomy of the joint and its soft tissue stabilizers, even small injuries can cause adhesion or misalignment among the structures, which can cause aberrant wear and stiffness. However, delayed treatment, as in this case of chronic PIP joint dislocation, may increase the risk of complications. This is in accordance with other case report conducted by Bamal, et al., where case of chronic PIP joint dislocation has reported open reduction via a dorsal approach [7]. They believe that a midaxial approach provides extensile exposure to the PIP joint, allows adequate soft tissue release, and it is possible to visualize and reduce the articular surfaces. On this case we performed with open reduction and soft tissue repair of chronic PIP joint dislocations using a zig-zag approach. Their outcome can successfully achieve a functional range of motion with a stable joint but leave a dorsal scar when flexing the finger in the early postoperative period but on this case scar on the volar may be less painful than dorsal scar.

There is no consensus on the best treatment approach for PIP joint dislocation. In addition, evidence suggests that post-treatment outcomes rarely return a full AOM [5]. However, the treatment plan must focus on the rehabilitation of all damaged components, including osseous, articular, and soft tissue structures to achieve optimum results [6]. In this case, open reduction of the chronic PIP joint dislocation successfully produced a functional range of motion with a stable joint. Rehabilitation in case of volar dislocation, distal interphalangeal motion is encouraged in the early postoperative period and PIP joint motion is commenced at 2 weeks, allowing active flexion and full range of motion encouraged after that. This case report has been reported in accordance with the Surgical Case Report (SCARE) 2023 Criteria [8].

## 4 Conclusion

A neglected case is a challenging case for reconstruction and repair, especially for the joint and articular cartilage. This case report demonstrates that even in cases of chronic, neglected PIP joint dislocations, surgical intervention with open reduction, collateral ligament reconstruction, and volar plate repair can successfully restore a functional range of motion and joint stability, provided the articular cartilage remains relatively preserved. Early intervention in PIP joint dislocations is crucial to minimize complications and optimize functional outcomes. However, this case highlights the potential for successful surgical management even in delayed presentations, offering hope for patients with similar complex injuries.

## Author contribution

Heri Suroto involved in performing surgical technique, conceptualization, investigation, project administration, resources, supervision, validation, writing-original draft, writing-review & editing.

Yohanes Aprianto Senduk Widodo involved in performing surgical technique, formal analysis, visualization, writing-original draft, writing-review & editing.

## Informed consent

Appropriate consent was obtained from all individual participants included in the study.

## Ethical approval

Regarding to the observational study of outcome in our case report, the ethical approval was waived by an institutional review board. However, the copies of informed consent are available for review by the Editor-in-Chief of this journal on request.

## Guarantor

Heri Suroto.

## Research registration number

Not applicable.

## Funding

None.

## Conflict of interest statement

The authors have no conflicts of interest to disclose.
